# Utility of malignancy markers in fine-needle aspiration cytology of thyroid nodules: comparison of Hector Battifora mesothelial antigen-1, thyroid peroxidase and dipeptidyl aminopeptidase IV

**DOI:** 10.1038/sj.bjc.6604194

**Published:** 2008-01-22

**Authors:** C de Micco, V Savchenko, R Giorgi, F Sebag, J-F Henry

**Affiliations:** 1 Laboratoire d'Anatomie et de Cytologie Pathologique, Faculté de Médecine, Bd Pierre Dramard, 13916 Marseille Cedex 20, France; 2 Laboratoire de Biochimie Endocrinienne et Métabolique (EA 3288), Faculté de Médecine, Université de la Méditerranée, 27 Bd Jean Moulin, 13385 Marseille cedex 5, France; 3 Center of Endocrine Surgery, Divisions of Endocrinology and Oncology, 121 Kharkivske Shosse Kiev, 252000 Ukraine; 4 Laboratoire d'Enseignement et de Recherche sur le Traitement de l'Information Médicale, Faculté de Médecine, Université de la Méditerranée, 27 Bd Jean Moulin, 13385 Marseille cedex 5, France; 5 Departement de Chirurgie Générale et Endocrinienne, CHU La Timone, 264 Rue Saint Pierre, 13005 Marseille, France

**Keywords:** Hector Battifora mesothelial antigen-1, thyroid peroxidase, dipeptidyl aminopeptidase IV, fine-needle aspiration cytology, thyroid nodule

## Abstract

The purpose of this study was to compare the diagnostic interest of Hector Battifora mesothelial antigen-1 (HBME-1), thyroid peroxidase (TPO), and dipeptidyl aminopeptidase IV (DPP4) in thyroid fine-needle aspirates obtained from 200 resected thyroid lesions (55 colloid nodules, 54 follicular adenomas, 59 papillary cancers, and 32 follicular carcinomas). Hector Battifora mesothelial antigen-1 or TPO expression (% positive cells) and DPP4 staining score (12-point scale) were evaluated. Receiver operating characteristic (ROC) curves were plotted and optimal cutoff values for diagnosing malignancy were determined. The TPO ROC curve was consistently higher than the HBME-1 ROC curve. The TPO curve was also higher than the DPP4 curve with regard to sensitivity, but dipped below the DPP4 curve with regard to specificity. Using a cutoff value of <80% positive cells for TPO, >10% positive cells for HBME-1, and staining score ⩾1 for DPP4, sensitivity to specificity ratios were 98–83% for TPO, 90–60% for HBME-1, and 88–80% for DPP4. Two particularly interesting findings of this study were the low negative likelihood ratio of TPO (0.02) allowing highly reliable exclusion of malignancy and the 100% specificity of DPP4 staining scores=12. Due to poor performance on follicular lesions, HBME-1 showed no advantage over TPO or DPP4.

Fine-needle aspiration (FNA) biopsy is the most effective method for preoperative evaluation of thyroid nodules (Cooper *et al*, 2006). This efficacy depends mainly on the fact that almost 70% of thyroid nodules are benign macrofollicular lesions that can be reliably identified in cytological smears, and that fewer than 5% are malignant tumours including 70% of papillary cancers (PCs) that can also be reliably identified on FNA. However, overlapping cytological features can hinder diagnosis in the remaining 25% cases. Fine-needle aspiration biopsy cannot distinguish benign from malignant microfollicular tumours that require examination on histological sections. It can be difficult to distinguish between hyperplastic nodules and follicular tumours and to recognize the follicular variant of PC (De Micco *et al*, 1999). Because of these drawbacks, FNA smears presenting microfollicular or hypercellular pattern are classified as follicular neoplasm, a ‘grey zone’ of thyroid FNA (Baloch *et al*, 2002).

Various methods have been proposed to assist thyroid cytology on FNA. Molecular methods such as mutation detection or expression profile analysis are promising but cannot currently be used for routine practice (Eszlinger *et al*, 2006; Griffith *et al*, 2006; Lubitz *et al*, 2006; Tetzlaff *et al*, 2006; Finn *et al*, 2007). On the contrary, immunocytochemistry (ICC) and cytoenzymology (CE) are widely available and can be performed on smears (De Micco *et al*, 1994a), cell blocks (Sack *et al*, 1997), or liquid-based preparation (Rossi *et al*, 2005). Out of several markers that have been investigated, the most attractive for diagnosis on thyroid FNA are Hector Battifora mesothelial antigen-1 (HBME-1), thyroid peroxidase (TPO), and dipeptidyl aminopeptidase IV (DPP4) because they can be used directly on smears.

Thyroid peroxidase is a membrane thyroid enzyme essential for thyroid hormone synthesis. It is present in large quantity in the cytoplasm of all benign thyrocytes. In malignant tumours, TPO synthesis is inhibited to varying degrees and maturation is deregulated resulting in overexpression of short splice variants (De Micco *et al*, 2000; Di Cristofaro *et al*, 2006). According to gene expression profiling analysis in thyroid cancer *vs* non-cancer tissue, TPO was one of the top 12 candidate markers in terms of diagnostic utility (Griffith *et al*, 2006). The TPO immunoreactivity is reduced in malignant cells especially in response to antibodies reacting with the native form such as the monoclonal antibody 47 (MoAb 47) (De Micco *et al*, 1991). Previous studies (De Micco *et al*, 1994a; Faroux *et al*, 1997; De Micco *et al*, 1998; Christensen *et al*, 2000) have confirmed the value of TPO ICC using MoAb 47 on smears.

Dipeptidyl aminopeptidase IV is an exopeptidase identical to cluster antigen CD26. Encouraging results have been obtained using DPP4 CE for diagnosis of thyroid malignancy on FNA material (Aratake *et al*, 1992; Zoro *et al*, 1996). A high-contrast red stain appears on the cellular membrane in malignant thyroid cells. Detection of DPP4 activity on smears is simple, fast, and inexpensive.

Hector Battifora mesothelial antigen-1 is a monoclonal antibody raised against cultured mesothelial cells. It reacts with an unknown antigen on the surface of mesothelial cells and in various adenocarcinomas and sarcomas (Miettinen and Karkkainen, 1996). Hector Battifora mesothelial antigen-1 immunohistochemistry is usually negative in benign thyroid lesions and positive on carcinomas. Most studies using HBME-1 for diagnosis of thyroid tumours have been performed on tissue samples (Mai *et al*, 2002; Ito *et al*, 2005; Papotti *et al*, 2005; Prasad *et al*, 2005). A few studies using FNA products have been carried out on cell blocks or thin layers (Sack *et al*, 1997; van Hoeven *et al*, 1998; Rossi *et al*, 2005; Saggiorato *et al*, 2005). We have developed an HBME-1 staining technique for air-dried thyroid FNA smears. The purpose of this report is to compare the utility of HBME-1, TPO, and DPP4 for diagnosing thyroid malignancy on FNA smears.

## MATERIALS AND METHODS

### FNA smears

Smears were made using FNA samples obtained from 200 thyroid lesions investigated and removed in the Department of Endocrine Surgery from 1993 to 2006 (Timone University Hospital, Marseilles, France). There were 55 benign colloid nodules (CNs), 54 follicular adenomas (FAs) including seven cases with limited nuclear features of papillary thyroid cancers (LNFPTCs) and six with Hürthle cells, 59 PCs including 19 follicular variants (PCFVs), and 32 follicular carcinomas (FCs) including 9 Hürthle cell carcinomas. None of these tumours were hyperfunctioning. Consecutive cases were selected from hospital files according to the WHO's classification-based registered histological diagnosis and to the availability of FNA material.

Pre-operative work-up included FNA in all patients. Thyroid peroxidase and DPP4 analysis were routinely performed on three slides (two TPO and one DPP4) as previously described (De Micco *et al*, 1994a; Zoro *et al*, 1996). The material was spread on super-frost slides (CML, Nemours, France), air dried, and either stored at +4°C to be stained within 72 h (100 cases) or frozen at −20°C for future staining (100 cases).

Hector Battifora mesothelial antigen-1 analysis and controls were performed on smears made from additional FNA obtained from the resected nodule in the surgery room, air dried, and immediately frozen and stored at −20°C.

This study protocol was approved by the clinical research committee of Marseille Public Hospital System.

### TPO and HBME-1 immunocytochemistry

After neutralisation of endogenous peroxidase in a phosphate-buffered saline (PBS) solution containing 0.1% hydrogen peroxide (H_2_O_2_) for 5 min, slides were incubated overnight at +4°C with anti-TPO monoclonal antibody (MoAb 47 clone; Dako, Glostrup, Denmark) diluted to 1 : 100 (2 *μ*g ml^−1^). Revelation was performed using a streptavidin–biotin–peroxidase kit with 3,3′-diaminobenzidine (LSAB; DAKO, Glostrup, Denmark). The percentage of positive cells was evaluated. Cells exhibiting an abnormal staining pattern, that is, low intensity with coarse granules at the periphery of the cytoplasm with no perinuclear ring, were not considered as positive. Positive controls were performed on smears from operated benign nodules. Negative controls were performed by omitting the primary antibody.

Primary anti-HBME antibody was used at a dilution of 1 : 100 (1 *μ*g ml^−1^) and the reaction was performed using an automated immunoperoxidase procedure in the Ventana Benchmark device (Ventana kit; Ventana, Tucson, AZ, USA). Results were evaluated without knowledge of the final histological diagnosis and that of the results of TPO and DPP4 reactions. Two independent observers (CDM and VK) evaluated the percentage of positive cells, and the final results corresponded to the mean of the assessments. Smears using the same techniques on PCs were performed as positive controls.

### DPP4 cytoenzymology

Detection of DPP4 activity was performed as follows:

- fixation for 1 min in a mixture of formalin, cold acetone, and PBS (0.15 mol l^−1^, pH 7.4) at a ratio of 1 : 40 : 10 respectively;
- rinsing with water;
- immersion for 30 min at room temperature in a solution containing 3 mg glycyl proline 4-b naphthylamide, 0.25 ml *N*,*N*-dimethylformamide, and 5 mg fast blue B salt in 4.6 ml sodium phosphate buffer (0.1 mol l^−1^, pH 7.2);
- rinsing with water;
- counterstaining with haematoxylin for 10 s and mounting with water.

Positive reactions are characterized by contrasted red staining of the cytoplasm and plasma membrane. Staining was scored using a discontinuous 0–12-point scale as described previously (Aratake *et al*, 1992). Positive controls were performed on smears from operated malignant nodules.

### Statistical analysis

SPSS software (version 13.0, 2004 SPSS Inc., Chicago, IL, USA) was used for statistical analysis. A *P*<0.05 was considered statistically significant. Receiver operating characteristic (ROC) curves were constructed to compare the diagnostic performance of the three markers and the area under the ROC curve (AUC) was computed (Hanley and McNeil, 1982). Cutoff values resulting in optimal sensitivity (Se) to specificity (Sp) ratios were determined based on the ROC curves. In addition to Se and Sp, positive and negative likelihood ratios (LR+ and LR−) were calculated.

## RESULTS

Thyroid peroxidase ICC and DPP4 staining performed on control smears obtained from nodules after surgical resection showed no significant differences as compared with pre-surgically obtained FNA samples. Indeed, the higher proportion of epithelial to red blood cells observed in post-surgical FNAs did not modify the pattern of staining of the markers or the ratio of positive to negative epithelial cells. Reactions for TPO, DPP4, and HBME1 performed on control slides stored at +4°C or frozen at −20°C for up to 10 years were also undistinguishable.

Results obtained with TPO, HBME-1, and DPP4, based on definitive histological diagnosis, are shown in Tables 1, 2. Overall, clearly positive or negative reactions were obtained in 146–163 of the cases depending on the marker used. However, intermediate reactions with overlapping of benign and malignant tumours were observed in other cases.

**Figure 1:**
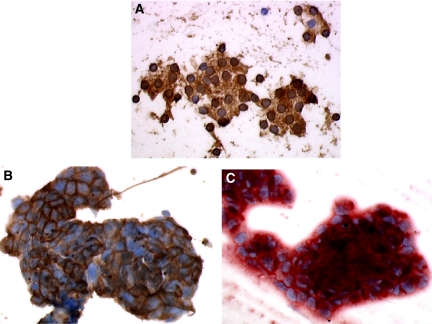
Pattern of positive staining of TPO, HBME, and DPP4. (**A**) Staining of TPO in benign follicular adenoma: brown granular deposit all over the cytoplasm and around the nuclei. (**B**) Staining of HBME in papillary cancer: brown reaction predominant on cellular membranes. (**C**) Staining of DPP4 in papillary cancer: contrasted red reaction within cytoplasm and along membranes.

Immunostaining of TPO was highly positive (⩾80% positive cells) in 90 (51 CNs and 39 FAs) out of the 109 benign nodules. The reaction was characterised by dark-brown granular staining of the cytoplasm with halo around nuclei (Figure 1A). Two nodules (one CN and one FA) were negative and 17 (three CNs and 14 FAs) exhibited varying percentages of positive cells. Three positive FAs contained LNFPTCs and four contained Hürthle cells. The TPO reaction was negative or low grade (⩽10%) in 52 out of the 91 carcinomas (41 PCs and 11 FCs) and positive (100%) in two PCs. Both positive PCs were PCFVs and one presented as a benign CN including foci of microcarcinoma. Intermediate TPO immunostaining (20–70%) was observed in the remaining 37 carcinomas, that is, 16 PCs and 21 FCs. Tall-cell tumours and tumours with Hürthle cell features exhibited an abnormal reaction pattern characterized by pale granular staining beneath the cellular membrane with absence of the nuclear halo.

Immunostaining of HBME-1 was negative or low grade (⩽10% positive cells) in 65 out of the 109 benign nodules (37 CNs and 28 FAs), and high grade (⩾80%) in 14 including five CNs and nine FAs. Intermediate staining (20–70%) was observed in the remaining 30 cases. Nine of the positive benign nodules exhibited LNFPTCs and six were made of Hürthle cells. Strong membrane staining sometimes associated with a diffuse cytoplasmic reaction involving more than 70% of cells was observed in 71 out of the 91 carcinomas (Figure 1B). Weaker intermediate staining (20–70% positive cells) was observed in 10 PCs and four FCs. Five PCs and four FCs showed low-grade staining (⩽10%). Four out of these five PCs were PCFVs and one contained multiple foci of microcarcinomas within a CN. Out of the four negative FCs, one was made of Hürthle cells, two were minimally invasive, and one was highly invasive and poorly differentiated.

Staining scores of DPP4 were negative or light (⩽1) in 87 out of the 109 benign nodules, including 45 CNs and 42 FAs, and clearly positive (>4) in seven FAs. Intermediate scores were obtained in the remaining 15 cases. Three positive benign nodules contained LNFPTCs and three were made of Hürthle cells. In the 91 carcinomas, scores ⩾6 were obtained in 62 cases (Figure 1C). However, 11 carcinomas (four PCs and seven FCs) had scores ⩽1. Three PCs were PCFVs, three FCs contained Hürthle cells, three were minimally invasive, and one was a poorly differentiated insular tumour.

**Figure 2:**
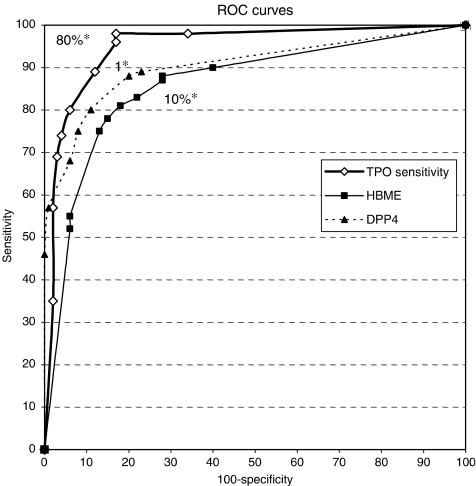
Receiver operating characteristic curves obtained with threshold values from 0 to 100% for TPO, 100 to 0% for HBME, and 12 to 0 for DPP4. ^*^Threshold values resulting in the best sensitivity for each marker.

To determine the best cutoff values for diagnosis of malignancy, ROC curves were constructed and analysed (Figure 2). The AUC was 0.95 (IC 95%: 0.92–0.98) for TPO, 0.90 (IC 95%: 0.86–0.95) for DPP4, and 0.86 (IC 95%: 0.81–0.92) for HBME. These differences were not significant. However, the TPO ROC curve was always entirely above the HBME-1 ROC curve and above the DPP4 ROC curve in the segment corresponding to high sensitivity. It crossed the DPP4 ROC curve to get under it in the segment corresponding to high specificity. Optimal Se to Sp ratios were observed using the following cutoff values for malignancy: ⩾1 for DPP4 staining score, >10% positive cells for HBME-1, and <80% positive cells for TPO. With these cutoffs, Se was 0.89 (IC 95%: 0.79–0.95) for DPP4, 0.90 (IC 95%: 0.82–0.95) for HBME-1, and 0.98 (IC 95%: 0.92–1) for TPO; Sp was 0.60 (IC 95%: 0.50–0.69) for HBME-1, 0.80 (IC 95%: 0.71–0.86) for DPP4, and 0.83 (IC 95%: 0.74–0.89) for TPO.

Calculation of LR+ and LR− yielded values of 2.5 and 0.17, respectively, for HBME-1; 5.8 and 0.02, respectively, for TPO; and 4.4 and 0.15, respectively, for DPP4. One nodule, that is, a multifocal micropapillary carcinoma within an adenoma, was false negative with all three markers. Three other nodules, that is, one PCFV and two minimally invasive FCs, were false negatives with HBME-1 and DPP4. Maximum Sp values were 0.97 (IC 95%: 0.92–0.99) with HBME-1, 0.98 (IC 95%: 0.93–1) with TPO, and 1 with DPP4, using cutoff values of ⩾90% positive cells for HBME, ⩽10% positive cells for TPO, and a score of 12 for DPP4. The Se with these cutoffs was 0.46 (IC 95%: 0.36–0.37) for DPP4, 0.55 (IC 95%: 0.44–0.65) for HBME-1, and 0.57 (IC 95%: 0.46–0.67) for TPO. One CN and five FAs were false positive with HBME-1 and TPO.

## DISCUSSION

Thyroid peroxidase ICC using MoAb 47 and DPP4-activation detection were the first malignancy markers reported to have a diagnostic significance on thyroid FNA smears (Aratake *et al*, 1992; De Micco *et al*, 1994a). In large series, TPO has been shown to have Se of 98% and Sp of 80% (Faroux *et al*, 1997; De Micco *et al*, 1998; Christensen *et al*, 2000). Dipeptidyl aminopeptidase IV is less sensitive, especially for FC, but more specific. As the staining method is simple to perform on air-dried smears and is inexpensive, we routinely carry out DPP4 analysis in conjunction with TPO ICC at our institution to assist thyroid FNA.

A number of other molecules have been described later on as malignancy markers for thyroid carcinoma. Three of them, that is, HBME-1, galectine-3 (GAL3), and cytokeratine-19 (CK19), have been tried on cytological material and are considered useful (van Hoeven *et al*, 1998; Saggiorato *et al*, 2005; Rossi *et al*, 2006). However, almost all these studies were performed on cell blocks or thin-prep samples because of poor results on conventional smears. At our institution, unsuitability for use on FNA smears has been a major drawback for the routine use of HBME-1, GAL3, and CK19 when compared with TPO and DPP4. Indeed, the need to perform cell blocks or thin-prep preparations in addition to smears introduced an excessive increase in both procedure cost and time, when two methods efficient on smears were already available. Recently, we successfully developed an HBME-1 staining technique on FNA smears using an automated immunoperoxidase procedure in the Ventana Benchmark device. Then, comparison with other markers performed on FNA smears became possible.

In the present series, we compared the performance of TPO, DPP4, and HBME-1 on FNA smears obtained from 200 thyroid nodules before or after surgical resection. We performed ROC analysis to compare the results and determine optimal cutoff values for diagnosis of malignancy (Hanley and McNeil, 1982). Positive and negative LR were calculated for a better estimation of the diagnostic value independent of the proportion of histological types of tumours in the series (Jaeschke *et al*, 1994).

Fine-needle aspiration biopsy performed on resected thyroid nodules represents a useful tool for the analysis of molecular markers. Except for varying amount of blood, the properties of smears taken before and after surgical resection are basically identical. In our experience, thyroid FNA stored at −20°C for more than 10 years retained their morphology and immunostaining properties for all the investigated antigens (thyroglobulin, thyrocalcitonin, thyroid transcription factor 1, TPO, and HBME-1), provided that they were performed and frozen immediately after resection. As our results of HBME ICC on FNAC obtained on resected nodule are very similar to those previously reported on FNAC obtained percutaneously (Sack *et al*, 1997; Rossi *et al*, 2005), it is reasonable to assume that these differences in FNA sampling methods had no significant influence on ICC.

Results with TPO were similar to those in previous studies (De Micco *et al*, 1994a, 1994b, 1999; Faroux *et al*, 1997; Christensen *et al*, 2000). Optimal performances for diagnosing malignancy, with Se of 98% and Sp of 83%, were obtained using a cutoff value of <80% positive cells. All FCs and 72% of FAs were correctly diagnosed. Almost half of the false-positive results concerned follicular tumours with either LNFPTC or Hürthle features. This finding corroborates molecular studies showing that these borderline tumours may represent an intermediate form between benign and malignant tumours (Vasko *et al*, 2004). Two false negatives were heterogeneous FVPTCs occurring in benign CNs. The errors were probably due to inadequate sampling, resulting in discrepancies from slide to slide.

As previously reported (Zoro *et al*, 1996), DPP4 was reliable for diagnosis of classical PCs, but the cutoff for diagnosing malignancy (⩾1) was lower. The high proportion of malignant tumours in this series may account for this difference. Sensitivity of DPP4 for malignant follicular tumours including the FVPTC was low with a misdiagnosis rate of 20–30%, especially with tumours presenting Hürthle or tall-cell features. This high false-negative rate reduces the utility of DPP4 for diagnosis of follicular tumours. Despite this limitation, we still consider DPP4 useful because positive results are highly specific.

To our knowledge, all but two previous studies of HBME on thyroid FNA (Sack *et al*, 1997; Rossi *et al*, 2005) were carried out on cell blocks (van Hoeven *et al*, 1998; Saggiorato *et al*, 2005). However, overall results obtained on fresh tissue, including our study, and cell blocks are rather concordant. The sensitivity of HBME-1 staining was excellent (>90%) for classical PC (van Hoeven *et al*, 1998; Ito *et al*, 2005; Rossi *et al*, 2005), disappointing for PCFV, and poor (<65%) for follicular and undifferentiated cancers (Cheung *et al*, 2001; Mai *et al*, 2002; Ito *et al*, 2005; Prasad *et al*, 2005; Saggiorato *et al*, 2005). The reported specificity of HBME-1 staining ranged from 70% to more than 90% (Cheung *et al*, 2001; Prasad *et al*, 2005; Saggiorato *et al*, 2005). Thyrotoxic hyperplasia was consistently negative (Rossi *et al*, 2006) whereas nodular goitres were positive in up to 17% of cases (Ito *et al*, 2005). Significant reactions were also reported in 25% of thyroiditis (Prasad *et al*, 2005), 5–10% of benign follicular tumours devoid of atypical features, and up to 65% with LNFPTC or Hürthle cell features (Mai *et al*, 2002; Papotti *et al*, 2005; Prasad *et al*, 2005; Scognamiglio *et al*, 2006). The fact that specificity in our study was in the lower range may be due to a high proportion of atypical or Hürthle cell tumours and absence of hyper-functional nodules.

Receiver operating characteristic analysis showed that all three markers improved the performance of thyroid FNA, with AUCs above 0.8 in comparison with values between 0.58 and 0.74 obtained in a recent analysis in which performance of 11 cytologists was measured using AUC (Raab *et al*, 2006). Comparison of the three ROC curves indicated that the sensitivity of TPO was superior to that of HBME-1 and DPP4 and its specificity was also superior to that of HBME-1. This difference was mainly due to the greater ability of TPO to differentiate benign from malignant follicular proliferations, including PCFV and Hürthle cell tumours. As a result, LR− was significantly lower for TPO ICC (0.02) than either DPP4 (0.12) or HBME-1 (0.17). According to Jaeschke *et al* (1994), LR− values of less than 0.1 are highly significant, whereas values of 0.1–0.2 are only moderately conclusive.

It should be noted that a previous study comparing the diagnostic value of HBME1 and TPO on cell blocks yielded different results, that is, lower Se (80%) and higher Sp (96 and 86%, respectively) for both markers (Saggiorato *et al*, 2005). Several possible explanations can be proposed for this difference. The cell-block methodology may not have been sensitive enough for precise determination of the percentage of positive cells. Evaluation of immunostaining was semiquantitative and ROC analysis was not used to compare marker performance. Cutoff values for the diagnosis of malignancy were determined empirically. The cutoff value retained to separate benign from malignant tumours based on TPO ICC in the study by Saggiorato *et al* was 50% of positive cells, whereas the cutoff value determined to provide the best results in previous ROC analysis as in the present study was 80% of positive cells (Faroux *et al*, 1997).

This study comparing malignancy marker staining on smears from thyroid FNA confirms our previous findings that TPO ICC is the most sensitive method for diagnosis of malignancy and enhances the specificity of standard cytology. Hector Battifora mesothelial antigen-1 ICC provides no advantage over TPO and assessment of the staining pattern is more difficult. Although its sensitivity is low, detection of DPP4 activity presents several advantages including 100% specificity for staining scores of 12.

## Figures and Tables

**Table 1:** Thyroid peroxidase and HBME immunocytochemistry on FNA smears from 200 thyroid tumorss

**% of positive cells**												
**Histology**	**Ab**	**0**	**10**	**20**	**30**	**40**	**50**	**60**	**70**	**80**	**90**	**100**
CN (*n*=55)	TPO	1	0	1	0	0	2	0	0	5	3	43
	HBME	32	5	8	0	3	1	1	0	4	0	1
FA (*n*=54)	TPO	1	0	0	1	3	4	5	1	13	3	23
	HBME	19	9	5	0	4	4	2	2	4	1	4
PC (*n*=59)	TPO	28	13	4	0	2	6	2	2	0	0	2
	HBME	3	2	2	1	2	1	2	2	13	2	29
FC (*n*=32)	TPO	4	7	7	4	4	2	4	0	0	0	0
	HBME	4	0	0	0	1	1	1	1	5	1	18

CN=colloid nodule, FA=follicular adenoma, FC=follicular carcinoma, FNA=fine-needle aspiration, HBME=Hector Battifora mesothelial antigen, PC=papillary carcinoma.

The table presents the number of cases.

**Table 2:** Scores of DPP4 activity according to histological diagnoses

	**Staining scores**							
**Histology**	**0**	**1**	**2**	**3**	**4**	**6**	**9**	**12**
CN (*n*=55)	43	2	8	2	0	0	0	0
FA (*n*=54)	41	1	2	1	2	6	1	0
PC (*n*=59)	4	0	1	2	1	7	7	37
FC (*n*=32)	6	1	6	3	5	3	3	5

CN=colloid nodule, DPP4=dipeptidyl aminopeptidase IV, FA=follicular adenoma, FC=follicular carcinoma, PC=papillary cancer.

The table presents the number of cases.
